# A time evolving online social network generation algorithm

**DOI:** 10.1038/s41598-023-29443-w

**Published:** 2023-02-10

**Authors:** Pouyan Shirzadian, Blessy Antony, Akshaykumar G. Gattani, Nure Tasnina, Lenwood S. Heath

**Affiliations:** Department of Computer Science, Virginia Tech, Blacksburg, VA 24061 US

**Keywords:** Computational science, Computer science

## Abstract

The rapid growth of online social media usage in our daily lives has increased the importance of analyzing the dynamics of online social networks. However, the dynamic data of existing online social media platforms are not readily accessible. Hence, there is a necessity to synthesize networks emulating those of online social media for further study. In this work, we propose an epidemiology-inspired and community-based, time-evolving online social network generation algorithm (EpiCNet), to generate a time-evolving sequence of random networks that closely mirror the characteristics of real-world online social networks. Variants of the algorithm can produce both undirected and directed networks to accommodate different user interaction paradigms. EpiCNet utilizes compartmental models inspired by mathematical epidemiology to simulate the flow of individuals into and out of the online social network. It also employs an overlapping community structure to enable more realistic connections between individuals in the network. Furthermore, EpiCNet evolves the community structure and connections in the simulated online social network as a function of time and with an emphasis on the behavior of individuals. EpiCNet is capable of simulating a variety of online social networks by adjusting a set of tunable parameters that specify the individual behavior and the evolution of communities over time. The experimental results show that the network properties of the synthetic time-evolving online social network generated by EpiCNet, such as clustering coefficient, node degree, and diameter, match those of typical real-world online social networks such as Facebook and Twitter.

## Introduction

Most real-world networks can be categorized as time-evolving networks where the nodes and the edges are added or deleted over time. A well-known example of a time-evolving network is an online social network with nodes representing individuals or groups of individuals and edges representing interaction or connection between them. Analysis of social networks has been a widely researched domain since the 1930s^1^, covering problems as diverse as the spread of anomaly and fraud detection^2–7^, recommendation systems^8–12^, public health epidemics^13,14^, organizational and political behavior^15^, patterns of friendship and romantic relationships^16,17^, and criminology^18,19^. In a recent work, Li et al.^20^ proposed a model for dynamic community detection in temporal networks that exploits the topological structure of the networks at every time step at node level. Yasami et al.^21^ presented a statistical approach to detect anomalies in dynamic social networks by hypothesizing that the microscopic features of each node drive the network dynamics and gradually cascade to the neighboring nodes. Yao et al.^22^ established a model that predicts new links in a dynamic social network for a given time interval based on an earlier snapshot of the same network. The arrival of online social media platforms such as Facebook, Twitter, and Instagram invigorated the already existing interest in social networks and led to the desire to understand human relationships (at various levels like friendship, business, and professional), interaction and socialization patterns, and dissemination of information in a time-evolving context.

The study of the characteristics of online social networks, on the other hand, presents several difficulties. First, as these networks are extremely large, with billions of nodes and trillions of edges in prominent online social media platforms^23^, it is clear that producing and storing a high-quality real-time network from these platforms requires large amounts of computational resources. Additionally, due to data privacy and corporate policies, social media firms are generally unwilling to share data from their networks for research purposes. Further, even if a network is recorded and used to learn characteristics about a particular online social network, it is more probable that the data will be out of date by the time the study results are published or when the learned model is used to make network predictions. Another prominent challenge associated with social media data is that its quality is degraded due to noisy, unreliable, and missing data^24^. Given the constraints of acquiring and sharing dynamic online social networks, it has become essential, for the purposes of research, to computationally generate networks that resemble online social networks in terms of network properties. Such synthesized complex networks enable more robust online social network research.

### Related work

A number of algorithms exist for generating various time-evolving networks, including social networks^25–28^. There are three papers^29–31^ that present algorithms for generating time-evolving online social networks, each focusing on generating random networks under certain assumptions that are believed to hold true in online social networks. Yousuf and Kim^29,30^ have presented algorithms that evolve a model based on the ideas of the rich-get-richer, socialization over time, and the transitive nature of relations between nodes (individuals) in a social network. The model starts with a closed triplet as the initial network and then adds a single node along with some number of edges using a local preferential attachment rule to create closed friendship triangles at every time step. In another work, Luo et al.^31^ present an algorithm to evolve a small seed network to generate a series of static networks at different time steps through addition or deletion of nodes and edges at random, while preserving the community structure of the original seed network. In this work, we propose a model that focuses on individual node behavior as well as communities while evolving in time steps without being dependent on any network properties. Besides, our model differs from the existing works by providing tunable parameters to influence the evolution of the network over time. This parameterization enables the user to define the desired characteristics of the generated networks such as the rate of new users entering the social network at each time step, number of communities, volume of users in each community at different time steps, and evolution of edges between the nodes in the network.

Statistical analysis of the number of individuals on different online social media platforms indicates that the number of individuals over time follows a logistic curve^23,32^ and that the growth rate of the number of individuals decreases exponentially with time^33^. Furthermore, studies in marketing science demonstrate that the number of buyers of technology-related products follows a logistic curve^34–36^.

There has been extensive research into the network properties of real-world online social media platforms. Different studies demonstrate that the clustering coefficient of the Facebook network is approximately 0.16^37–39^. Similar studies indicate that the clustering coefficient of Microsoft Messenger, Orkut, and Youtube are 0.137, 0.171, and 0.136 respectively^40,41^. Studies on online social networks indicate that these networks, in general, have small diameter^38,41,42^. Furthermore, analysis of the degree distribution of online social networks such as those of Twitter and Facebook indicates that node degrees follow a long-tailed/ heavy-tailed distribution^38,40,41,43^.

### Our results

In this work, we present an epidemiology-inspired and community-based, time-evolving online social network generation algorithm (EpiCNet) that generates a graph $G^t=(V^t, E^t)$ for each time step *t* representing the synthetic network at that time, where $V^t$ represents the set of individuals (i.e., registered members) of the online social media at time *t* and $E^t$ represents the set of connections between the individuals’ accounts of the network at that time step. Furthermore, we keep track of a set of dynamic overlapping communities $C^t$ in the network and evolve these sets of communities at each time step. We show that the generated network at each time step has characteristics similar to those of real-world online social networks, such as the network of Facebook, in terms of number of nodes, average degree, degree distribution, clustering coefficient, community structure, and diameter. See section “Experimental results”.

Our algorithm includes a set of tunable parameters that influence the evolution of nodes, edges, and communities in the network. The changes in the properties of the generated networks with variation in the values of the tunable parameters are analyzed to select a final set of values that are employed to generate networks that resemble real-world online social networks. Generating networks with desirable network properties is intended primarily as a validation for EpiCNet.

### Our contributions

Most random network generation algorithms—dynamic or not—generate networks guided or constrained by properties concerning the overall network (e.g., modularity and clustering coefficient). In contrast, in our algorithm, the dynamics of the network is modeled on the behaviors of each individual, such as how many new neighbors they obtain over time, which individuals they pick to be their neighbours, and what rules they follow while deleting edges with neighbouring nodes. We model the evolution of each individual *v* at a given time step using its neighbor set and the communities that the individual *v* belongs to. In particular, each individual evolves independently.

Additionally, we exploit the notion of compartmental modeling from epidemiology^44^ in characterizing addition and deletion of nodes from the online social networks. This novel use of compartmental modeling makes our model readily interpretable and enables a time-dependent flow of individuals into and out of the synthetic network.

In our model, we consider overlapping community structures while creating the networks as it will help in generating more realistic networks. In the overlapping community structure, each individual is potentially a member of multiple communities. Our algorithm updates the connections of each individual in the synthetic network at each time step based on the set of communities in which the individual participates.

Finally, time is an essential aspect of time-evolving networks. Each node and the network as a whole evolve differently as the network matures over time; evolutionary characteristics themselves are time dependent. Our algorithm, unlike all previous ones, includes the time step as a parameter when defining the model functions that control the evolution of the network.

## Preliminaries

An online social network can be modeled as a graph $G=(V,E)$, where the set of nodes *V* consists of all the individuals in the online social media and each edge $(u,v)\in E$ between nodes *u* and *v* represents a platform specific relationship between the user accounts associated with those individuals in the online social media platform. The network *G* can be directed or undirected depending on the platform. Directed networks depict platforms where individuals follow each other, such as Instagram and Twitter, and the indegree (respectively, outdegree) represents the number of followers (respectively, followings). On the other hand, undirected networks are useful for social media platforms where connections are a type of relationship such as friendship in Facebook and LinkedIn. In this work, we focus first on undirected networks and extend the algorithm to directed networks in section “Extension to directed networks”.

### Compartmental models

Compartmental modeling^44–47^ is a technique in mathematical epidemiology to capture the population dynamics of an infection. The population of individuals is divided and assigned to different labelled compartments, with individuals moving between compartments as their state changes. A compartmental model is a directed graph in which each compartment is a node and each edge signifies a potential transition of individuals from one compartment to another. In epidemiology, these models are often expressed using ordinary differential equations but can also be defined using a stochastic framework. This model can be applied in the context of an online social network where the population is divided into various compartments with individuals moving from one compartment to another over time.

### Communities

A community in a network is a designated set of nodes that are typically highly connected. This community structure is one of the critical features that characterize real-world online social networks^48,49^. A network can have multiple communities; these can be overlapping or non-overlapping. Often, the designated communities $C_1,C_2,\ldots ,C_k$ of *G* form a partition of *V*. An overlapping community structure of nodes of a network is a set of communities ${\mathscr {C}}=\{C_1,\ldots ,C_k\}$, where each node is in at least one community but may be in multiple communities. In this work, for each node *u*, we define ${\mathscr {C}}_u$ to denote the set of all communities that the node *u* participates in. Often, based on the structure and nature of an online social network, the overlapping community structure is more relevant than a non-overlapping community structure.

## EpiCNet

In this section, we present our algorithm, EpiCNet, for generating time-evolving online social networks. We address the case of undirected networks first. We start with an empty network representing the online social network at time $t=0$. For all time steps $t\ge 1$, our algorithm updates the network $G^{ t-1}$ and generates network $G^{t}$ where $G^{t}$ represents the online social network at time *t*. For each time step, the algorithm follows three phases: (1) node update, (2) community update, and (3) edge update. In the node update phase, we remove a subset of the individuals from the network and add a set of new individuals to the network. This phase is based on compartmental modeling. In the next phase, we update the set of communities by creating new communities and adding individuals to the existing communities. Finally, in the edge update phase, we add and remove edges between the individuals in the network.

The algorithm includes a set of tunable parameters, the values of which define the characteristics of the generated networks. Table 1 lists the tunable parameters of EpiCNet, along with the selected values for each parameter. The parameter *N* specifies the total population of individuals taken into account during the algorithm’s execution and $t^*$ specifies the total number of time steps captured during the execution. $p_1$, $p_2$, and $c_0$ are three parameters used to control the number of individuals in the network at each time step. In the community evolution phase, we use the parameter $\beta$ to control the number of communities, $s_c$ to determine the initial size of the new communities, and $p_3$, $p_4$, and $p_5$ to define the evolution of the communities over time. Specifically, an existing individual could join or leave a community with probabilities $p_3$ and $p_4$ respectively. Furthermore, $p_5$ is the probability of merging two communities as a result of the creation of a new edge. Finally, in the edge evolution phase, the parameters $n_e$ and $p_6$ denote the average number of new connections made by each individual and the probability of an individual removing an existing connection, respectively.

**Table 1:** Tunable parameters used in EpiCNet and a set of initial values for them.

	Parameter	Value	Meaning
General	*N*	100,000	Total population of individuals
	$t^*$	100	Total number of time steps
Node evolution	$c_0$	0.1	Parameters of the compartmental model
	$p_1$	0.04	
	$p_2$	0.04	
Community evolution	$\beta$	0.15	Ratio of the number of communities to the number of individuals
	$s_c$	100	Initial size of the communities
	$p_3$	0.15	Probability of an individual joining a new community
	$p_4$	0.05	Probability of an individual leaving one of their communities
	$p_5$	0.05	Probability of merging two communities as a result of an edge creation
Edge evolution	$n_e$	10	Average number of new edges created by an individual at each time step
	$p_6$	0.1	Probability of an individual removing an edge at each time step
	$p_7$	0.25	Probability of connecting to a famous individual (for directed networks only)

Next, we describe each phase of the algorithm, as well as the definition and usage of each tunable parameter.

### Node update

Based on how individuals are using a social media platform, they can be assigned to three compartments as follows:

- **Unassociated (***U***):** Individuals who have never created an account or used the platform.
- **Registered (***R***):** Individuals who have an account and are current members of the platform.
- **Deleted (***D***):** Individuals who once had an account in the past but have deleted their accounts and are no longer members of the platform.

The changes in these compartments over time are captured using a compartmental model. In our model, for efficiency reasons, we only store the size of the compartments rather than the individuals in each compartment. At each time step *t*, let $N_U^t, N_R^t$, and $N_D^t$ denote the number of individuals in the compartments *U*, *R*, and *D* at time *t*, respectively. For each time *t*, we define

$$\begin{aligned} {\mathscr {N}}^t:= (N_U^t, N_R^t, N_D^t) \end{aligned}$$

to represent the three compartments at time *t*. The transition of individuals from one compartment to another can occur in one of the following directions:

1. **From** *U* **to** *R*: The individuals who create an account for the first time and join the online social media platform.
2. **From** *R* **to** *D*: The individuals who delete their accounts on the platform.
3. **From** *D* **to** *R*: The individuals who rejoin the platform by creating a new account.

The probability of an individual moving from one compartment to another is given either by a time-independent constant or a function that depends on time.

In EpiCNet, the probability of an individual moving from compartment *R* to *D* is the constant $p_1$, and the probability of an individual moving from compartment *D* to *R* is the constant $p_2$. On the other hand, the probability of an individual moving from compartment *U* to *R* is a time dependent probability function $p_0(t)$, defined below. As discussed before, the number of individuals registered on the real-world networks forms a logistic curve over time. Hence, we define $p_0(t) = c_0f(\frac{t-c_1}{c_2})$, where $f(x)= \frac{e^x}{(1+e^x)^2}$ is the derivative of the logistic function^50^, $c_0$ is a constant, $c_1$ determines the time where the logistic curve has its highest derivative, and $c_2$ is a value determining the ratio of highest to lowest value of $p_0(\cdot )$ as $f(0)/f(-\frac{c_1}{c_2})$. In our selected model, we set $c_1=t^*/2$ to move the highest number of individuals from *U* to *R* at time $t^*/2$. Also, for any value *x* such that $|x|> 5$, *f*(*x*) is negligible compared to *f*(0); thus, in our selected model, we set $c_2=c_1/5$, and therefore, we define $p_0(t)$ as

$$\begin{aligned} p_0(t) = c_0 f\left( \frac{t-\frac{t^*}{2}}{\frac{t^*}{10}}\right) . \end{aligned}$$

**Figure 1:**
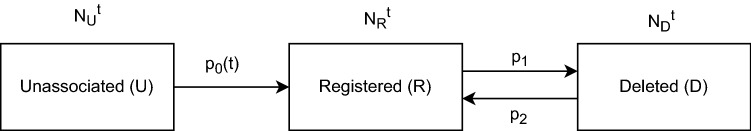
A schematic diagram of the compartmental model used for updating nodes of the network.

The compartmental model along with the transition of individuals between the compartments is represented in Fig. 1. The value associated with each edge in the compartmental model represents the probability of an individual moving from one compartment to another in the direction of the edge. Given that we only keep track of the compartment sizes, we use the binomial distribution to determine the number of individuals who moved from one compartment to another. More specifically,

1 $$\begin{aligned} N^t_{U\rightarrow R}\sim & {} B(N_U^{t-1}, p_0(t)),\nonumber \\ N^t_{R\rightarrow D}\sim & {} B(N_R^{t-1}, p_1),\\ N^t_{D\rightarrow R}\sim & {} B(N_D^{t-1}, p_2),\nonumber \end{aligned}$$

where *B*(*n*, *p*) represents a random value drawn from the binomial distribution with parameters *n* and *p*. Using the size of the compartments at time $t-1$ and the values defined on Equation (1), we determine the size of each compartment at time *t* as follows.

2 $$\begin{aligned} N_U^t= & {} N_U^{t-1} - N^t_{U\rightarrow R} \nonumber \\ N_R^t= & {} N_R^{t-1} - N^t_{R\rightarrow D} + N^t_{U\rightarrow R} + N^t_{D\rightarrow R}\\ N_D^t= & {} N_D^{t-1} - N^t_{D\rightarrow R} + N^t_{R\rightarrow D} \nonumber \end{aligned}$$

The node update process is formalized in pseudocode as shown below.
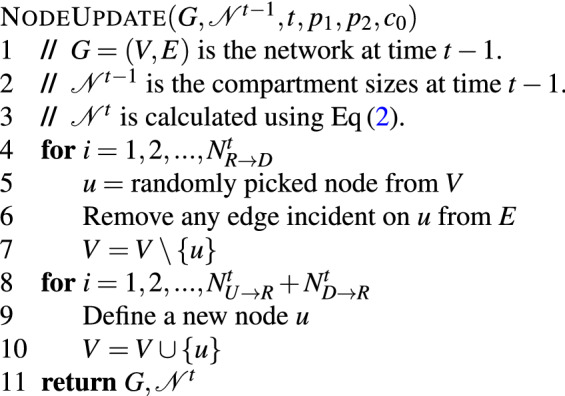


### Community update

The communities and the nodes belonging to each of them are tracked throughout the evolution of networks. At time step *t*, the network consists of $C^t$ communities and this number is linearly incremented as a function of time using the equation

$$\begin{aligned} |{\mathscr {C}}^t|\simeq \lceil \beta |N_R^t|\rceil , \end{aligned}$$

where $\beta \in [0,1]$ is a constant. EpiCNet allows a community to increase or decrease in size at every time step, through node and community evolution. However, the algorithm does not allow communities to explicitly split or merge. Since the creation and evolution of a community are done in a random manner, there is no unique attribute governing the members of the communities at any time step. A community ceases to exist when all of its constituting nodes are deleted. The evolution of a community may result in a complete change in its composition from its creation to deletion.

#### Creating new communities

At time step *t*, the number of new communities added in the network is

$$\begin{aligned} \max \left( 0,\left\lceil \beta \left( N_R^t-N_R^{t-1}\right) \right\rceil \right) . \end{aligned}$$

The newly added communities are initialized with $s_c$ nodes picked randomly from all the nodes in the network ($V^t$), where $s_c$ is a constant. This ensures that no community is created empty.

#### Evolving existing communities

Based on the dynamics of real-world online social networks, an individual may join an existing community or leave one of their communities every once in a while. To allow such events, at each time step *t*, every individual $v\in V$ is added to a randomly chosen community from ${\mathscr {C}}^t$ with probability $p_3$. Further, at each time step, every individual $v\in V$ could leave one of his/her communities with probability $p_4$. In the case of leaving a community event, we randomly choose one of their communities $C \in {\mathscr {C}}_v^t$ and remove *v* from *C*. However, the existing connections between *v* and the individuals in *C* remain unaltered.

#### Deleting communities

If all nodes in a community are deleted at time step *t*, then that community is deleted as well.

#### Merging communities

As observed in real-world social networks^51,52^, the evolution of the community structure in the network includes the occasional merger of two or more existing communities to form a new community. Usually, these merging events stem from the creation of new connections between individuals in the network. Inspired by this real-world observation, in our model, we consider the possibility of a merge event whenever a new edge is created. Hence, the merging of communities in the synthetic online social networks is embedded in the edge update phase (section “Edge update”). For every new edge (*u*, *v*) added between individuals *u* and *v*, the pair of individuals (*u*, *v*) is selected for a merge event with probability $p_5$. If selected, we pick two random communities $C_u \in {\mathscr {C}}_u^t$ and $C_v \in {\mathscr {C}}_v^t$ of *u* and *v* respectively where ${\mathscr {C}}_u^t$ denotes the set of communities that the individual *u* belongs to at a given time step *t*. Finally, the two communities, $C_u$ and $C_v$, are merged into a single community *C*.

The pseudocode below states the CommunityUpdate algorithm for the second phase.
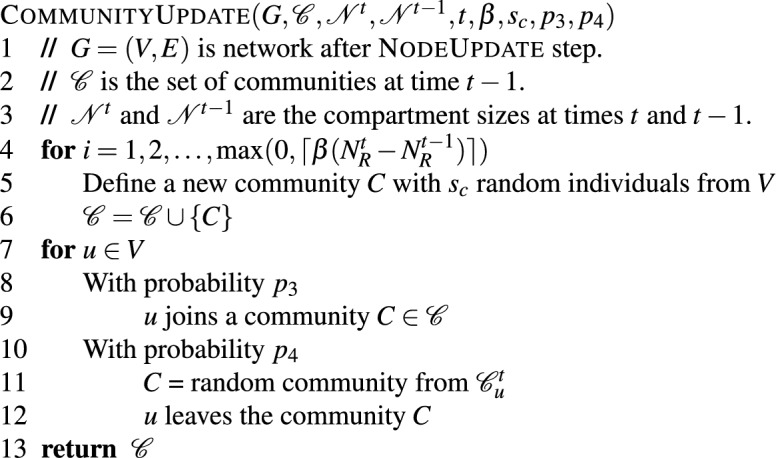


### Edge update

The edge update phase first does edge addition and then edge deletion.

#### Edge addition

The creation of edges between nodes is based on the hypothesis that individuals in an online social network tend to make more friends/connections with members of a community they have been recently added to than their older communities. At every time step *t*, every node *v* in $V^t$ creates $n_e$ new edges. Let ${\mathscr {C}}^t_v=\{C_1,\ldots ,C_k\}$ represent the set of communities node *v* is a part of at time *t* and let $t_i$ for $i\in [1,k]$ denote the time step at which the node *v* joined the community $C_i$. To add an edge for node *v*, a community $C_i$ is chosen from ${\mathscr {C}}^t_v$ in a weighted random manner with a weight proportional to $\frac{1}{(t-t_i)+1}$, where $(t-t_i)$ denotes the age of node *v* in the community $C_i$. In other words, a community to which *v* was recently added is given higher preference to create a new edge. A node $u\ne v$ is selected uniformly at random from the set of nodes in the chosen community. If an edge does not already exist between nodes *u* and *v*, then a new edge (*u*, *v*) is created and added to *G*.

#### Edge deletion

At time step *t*, every node *v* in $V^t$ is chosen for edge deletion with probability $p_6$, where $p_6\le 1$ is a constant. This is based on a hypothesis that not every individual deletes one of his/her connections at every time step. If *v* is selected for edge deletion, then a node *u* is picked uniformly at random from the set of neighbors of *v* and the edge (*u*, *v*) is deleted from the network.

The edge update pseudo-code is provided below.
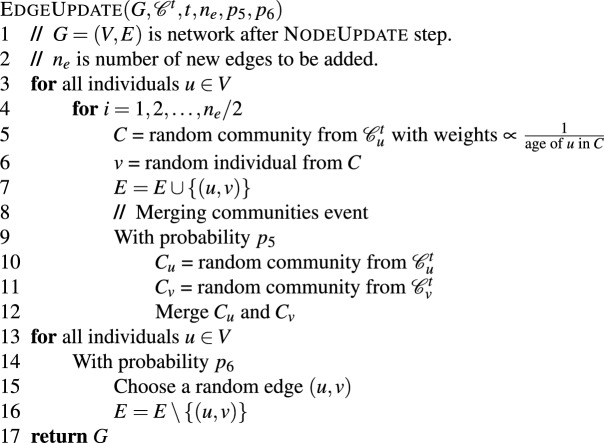


Using the three algorithms defined for the three phases, the overall steps of EpiCNet are shown in the form of a pseudo-code below.
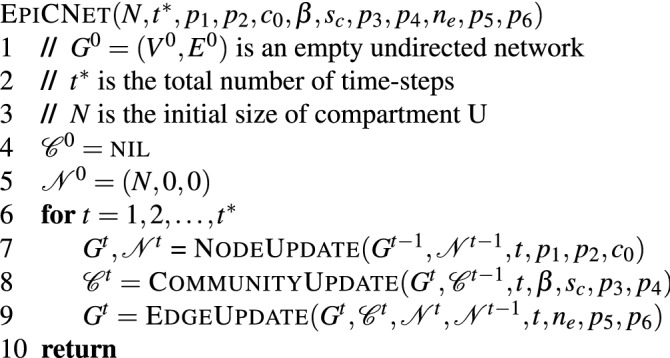


## Extension to directed networks

In this section, we provide a variant of our algorithm that produces random time-evolving directed online social networks. As mentioned earlier, a social media platform that allows for directed connections between individuals can be modeled using a directed network, in which the nodes stand in for the individuals, and any directed edge linking nodes *u* and *v* signifies a relationship between the user account associated with *u* and the user account associated with *v*. If a directed edge (*u*, *v*) exists in a network, we say that *u* (resp. *v*) is one of *v*’s (resp. *u*’s) followers (resp. followings). We employ a paradigm similar to the one described previously for creating undirected networks. The procedures for updating nodes and communities are identical to those for undirected networks, as the type of connections has no effect on the structure of nodes and communities. On the other hand, we adjust the edge update step slightly, as follows.

In contrast to undirected online social networks, directed networks allow individuals to follow a variety of user accounts they do not know, such as celebrities and influencers. As a result, there are individuals with an abnormally large number of followers who also have a regular number of followings, which we refer to as famous individuals. To account for this phenomenon, we introduce a new parameter, $p_7$, that defines the probability of creating a link to a famous individual. At each step in the edge update phase, for any individual *u* and for any edge to be created from *u* to another individual, with probability $p_5$, we choose a random famous individual *f* from the whole network and add an edge from *u* to *f*. For the sake of simplicity, we make no distinction between famous and non-famous individuals in the implementation; thus, to choose a famous individual at random, we just utilize a preferential attachment to select an individual from all individuals. We weigh individuals proportionally to the number of followers they have and choose one at random. The procedure below details the changes made to the EdgeUpdate procedure.
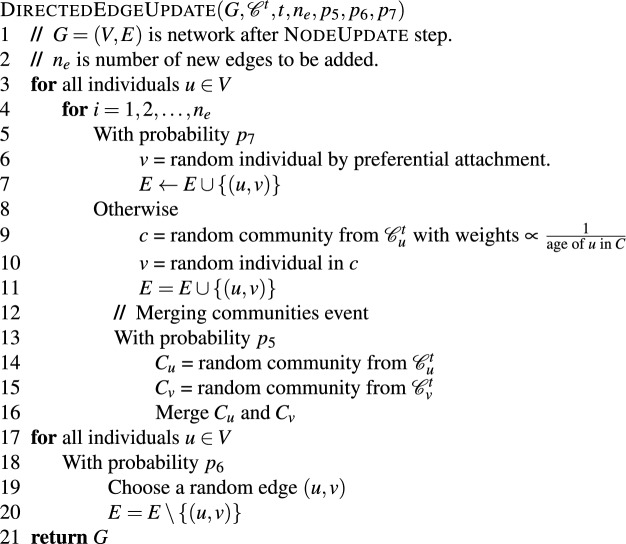


## Experimental results

This section contains details about the undirected and directed networks generated through the execution of the algorithm. The parameter values for generated networks were decided using hyperparameter tuning wherein we varied the different combinations of tunable parameters over a range of values to study the trends of various network properties over time.

### Results of the selected undirected model

Having studied the effects of different values for each of the tunable parameters, a final set of parameter values were selected to generate a sequence of time evolving networks that aligns with the real-world online social networks thereby verifying the hypotheses on which the proposed model is based. The final values of the parameters are listed in Table 1. This configuration of the network generator model was executed with $N = 100,000$ and for 100 time steps, which generated a sequence of networks with the below trends in network properties. The values of these properties in the final network are in Table 2.

**Table 2:** Properties of the generated networks of the selected undirected model.

Parameter	Value
$N_R^{t^*}$	32,355
Number of Edges	2,091,277
Diameter	6
Average degree	129.2
Clustering coefficient	0.30

Next, we analyse the characteristics of the synthetic online social networks.

#### Number of individuals and connections

**Figure 2:**
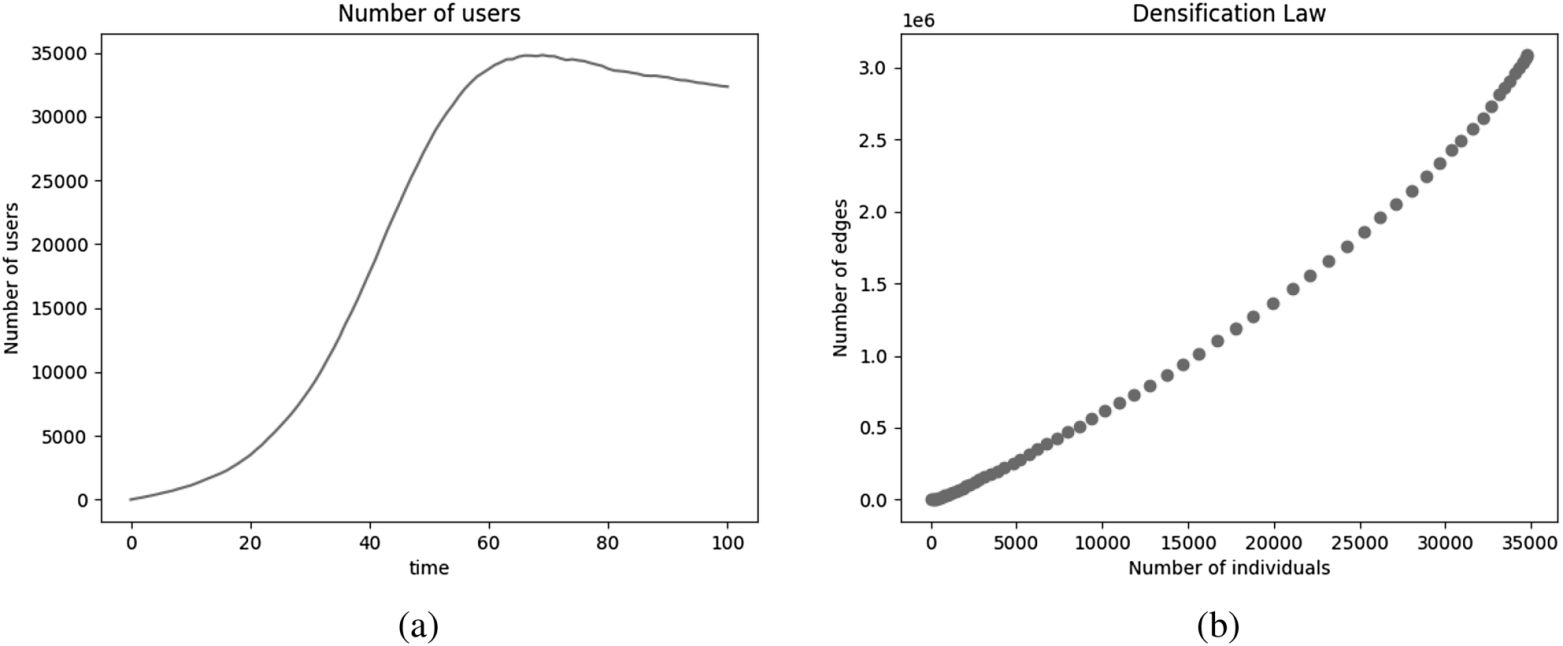
(**a**) Number of individuals and (**b**) number of edges of undirected networks generated using selected model.

The number of individuals being added to the network from a predefined population is seen in Fig. 2a. We observe a steep increase in the number of new individuals signing on to the online social network in the first half of the time frame and slowly this number stabilizes for the latter half. This validates the studies^23,33^ stating that the growth in the number of users of a new social media application decreases exponentially with time, eventually leading to a saturation in the number of individuals. This convergence of network size is achieved using the first two compartments in the model, i.e., Unassociated (*U*) and Registered (*R*) and the rules defined to update these compartments.

**Figure 3:**
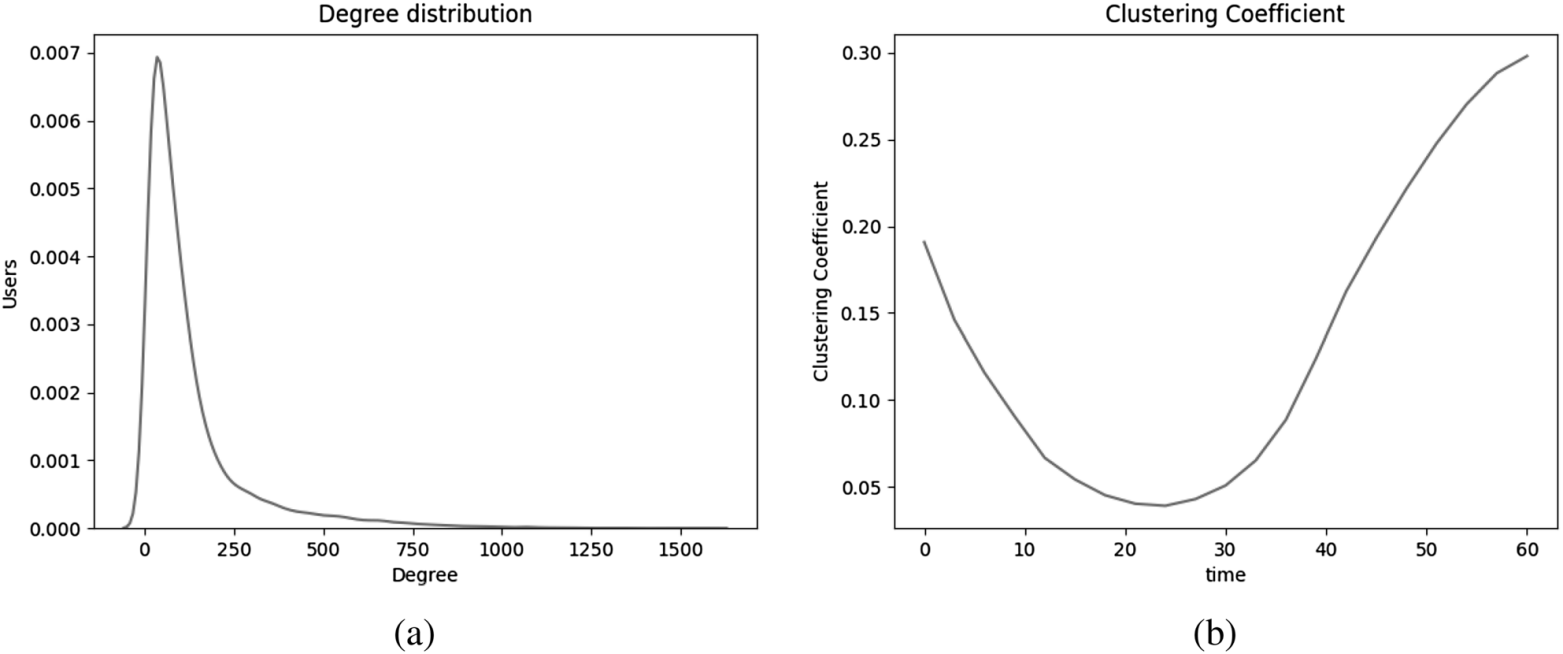
(**a**) Degree distribution and (**b**) clustering coefficient of undirected networks generated using selected model.

Figures 2b and 3a give the bigger picture about the number of individuals a user account is connected to. In Fig. 2b, we see a super-linear increase in the number of edges proportional to the number of individuals in the network. This phenomenon where the number of edges grow super-linearly in the number of nodes of the network is known as the densification law and is observed in the real-world networks^53^. The degree distribution exhibits a long-tailed/ heavy-tailed distribution as also seen in real-world online social networks of Flickr, LiveJournal, Orkut, Youtube, Google+, and Twitter^41,43,54^.

#### Clustering coefficient

Figure 3b shows the trends of clustering coefficient over time. The clustering coefficient of newly added users is smaller than that of the existing users. Thus, the global clustering coefficient of the network depends on the rate at which new users are added to the network. At all times, the clustering coefficient of the network varies in the range of [0.05, 0.30], which aligns with the clustering coefficient in applications like YouTube: 0.13, Facebook: 0.16, Orkut: 0.17, Twitter: 0.19, and Flickr: 0.31^38,41,43^.

#### Communities

**Figure 4:**
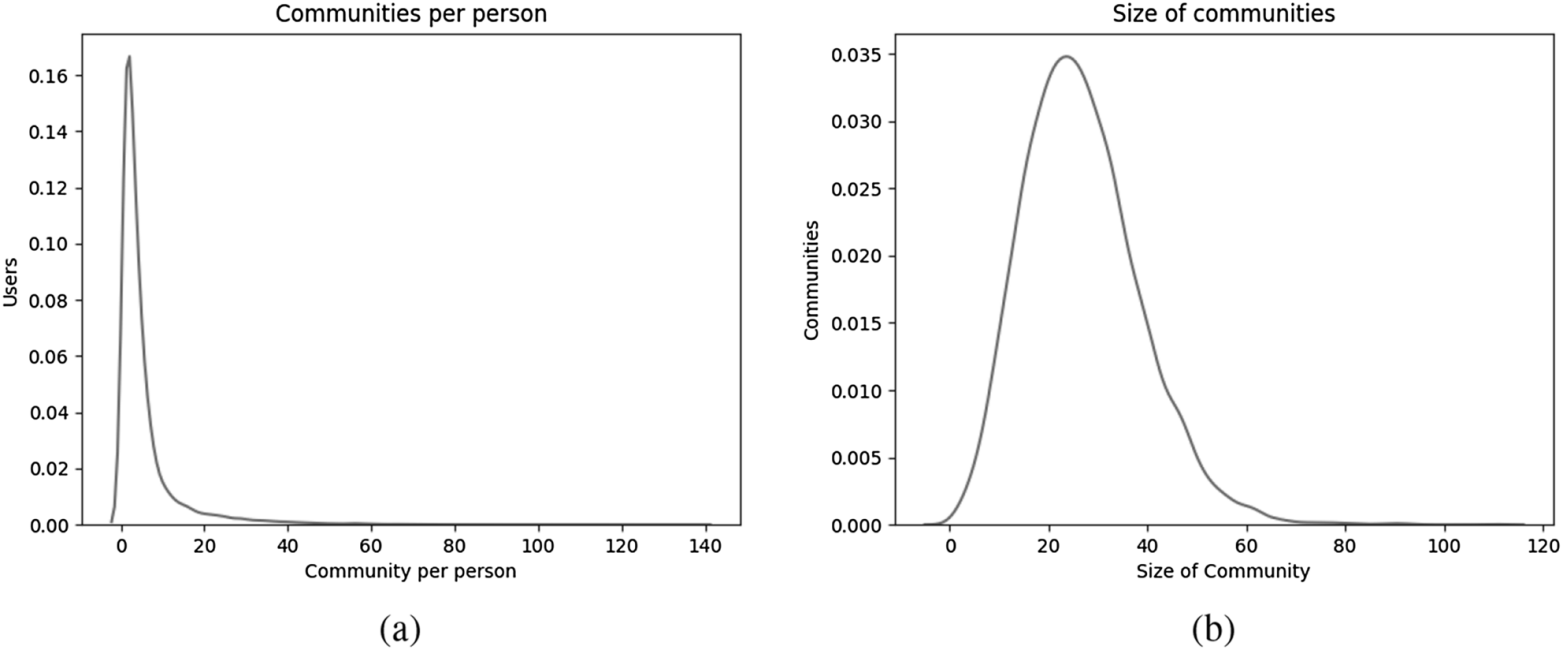
(**a**) Communities per individual and (**b**) size of communities of undirected networks generated using selected model.

In the real world, an individual is a part of one or more communities^49^ and this phenomenon is portrayed in the networks generated by EpiCNet. Figure 4a suggests that most of the individuals are in fewer than 20 communities with more than half of them in at most 10 communities. This is in line with the report on Facebook groups and their impact published in 2021^49,55^, which states that 1.8 billion individuals are a part of one or more groups with more than half of them in 5 or more groups.

The community size distribution in Fig. 4b is also consistent with observations in Facebook data^56^ that the number of communities tails off with increase in size of the communities. This observation suggests the presence of a large number of smaller communities.

#### Diameter

**Figure 5:**
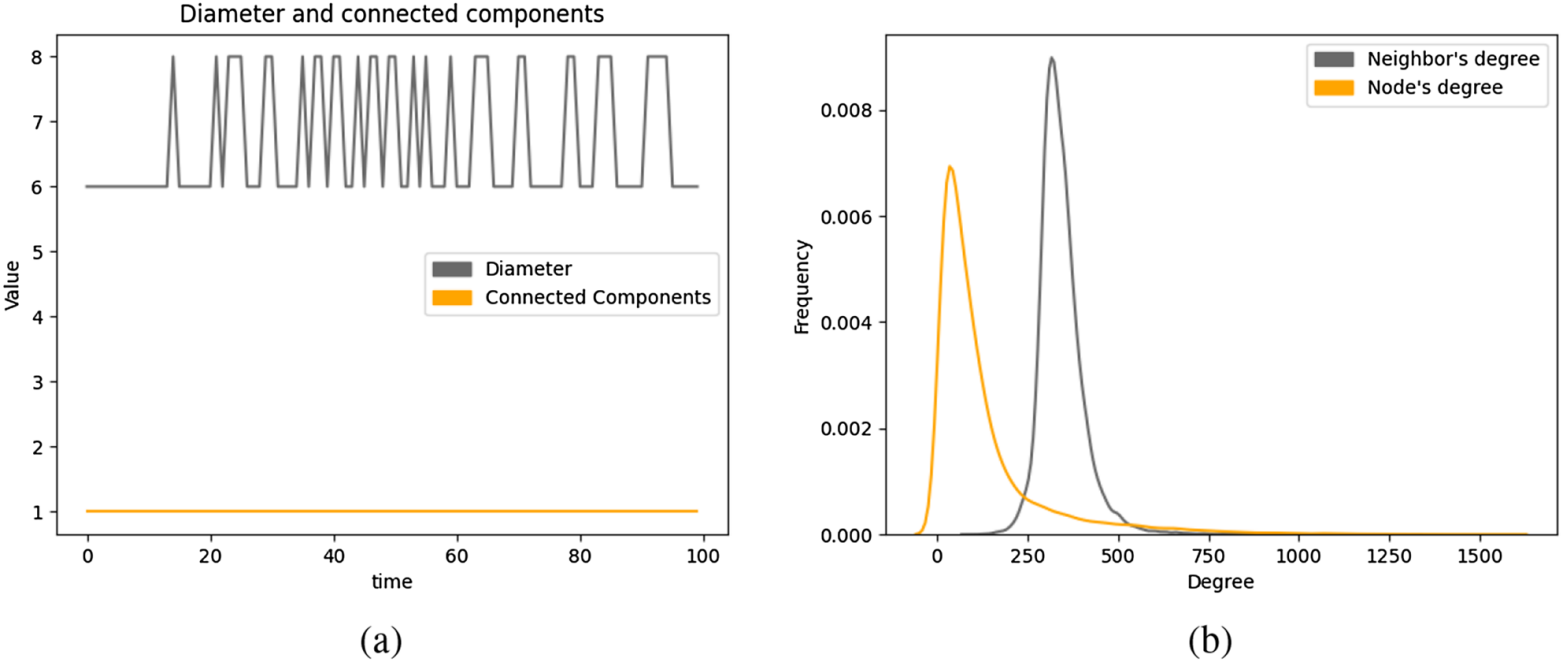
(**a**) Diameter and (**b**) average neighbor degree of undirected networks generated using selected model.

The network generated by our selected model is connected at all time steps. Figure 5a shows an upper-bound on the diameter of the network at all times, which is at most 8. Considering the diameter of online social media platforms, like Facebook discussed before, the diameter of the generated network is in line with that of real-world online social media platforms. Due to high time efficiency issues, we do not explicitly compute the exact diameter of the network. Instead, we compute the eccentricity of one random node *v* in our network, which is the maximum distance from *v* to any node in the network. Since the network is connected, the diameter of the network is at most two times the eccentricity of node *v*.

#### Friendship paradox

We compare the degree distribution of the node with the distribution of the average neighbor’s degree of the nodes, which is, for each node, the average degree of all its neighbors. Figure 5b shows that in expectation, the average neighbor’s degree of each node is higher than the degree of that node. This interesting characteristic is termed as the Friendship Paradox^57^, which states that the average number of friends of your friends is more than the number of your friends. The distributions of node degree and the average neighbor’s degree in Fig. 5b are normal distributions with different means. Since the mean value of the latter distribution is higher than the former, it is evident that for most of the nodes, the average neighbor’s degree is higher than its own degree. This behavior is also observed in the real world in the form of the Friendship Paradox phenomenon.

### Results of the selected directed model

**Figure 6:**
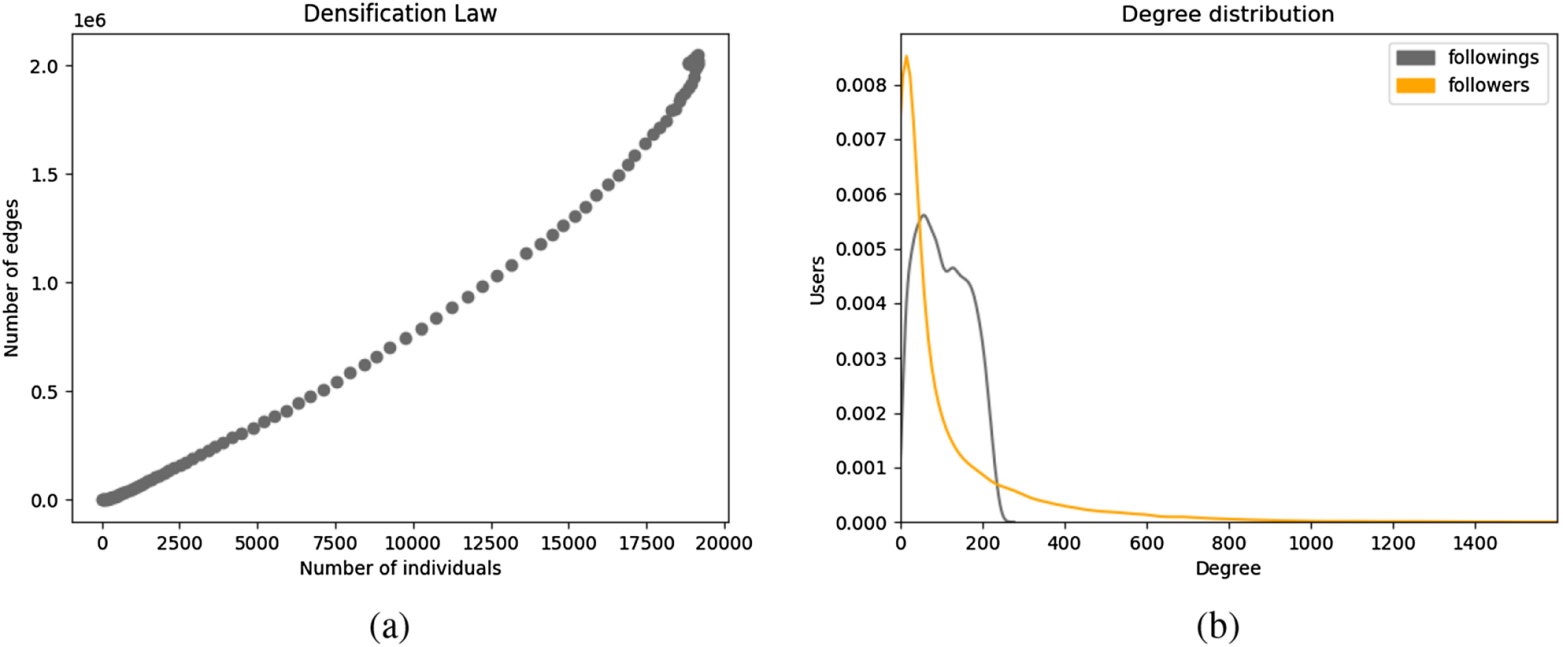
(**a**) Number of edges and (**b**) degree distribution of directed networks generated using the selected model.

**Figure 7:**
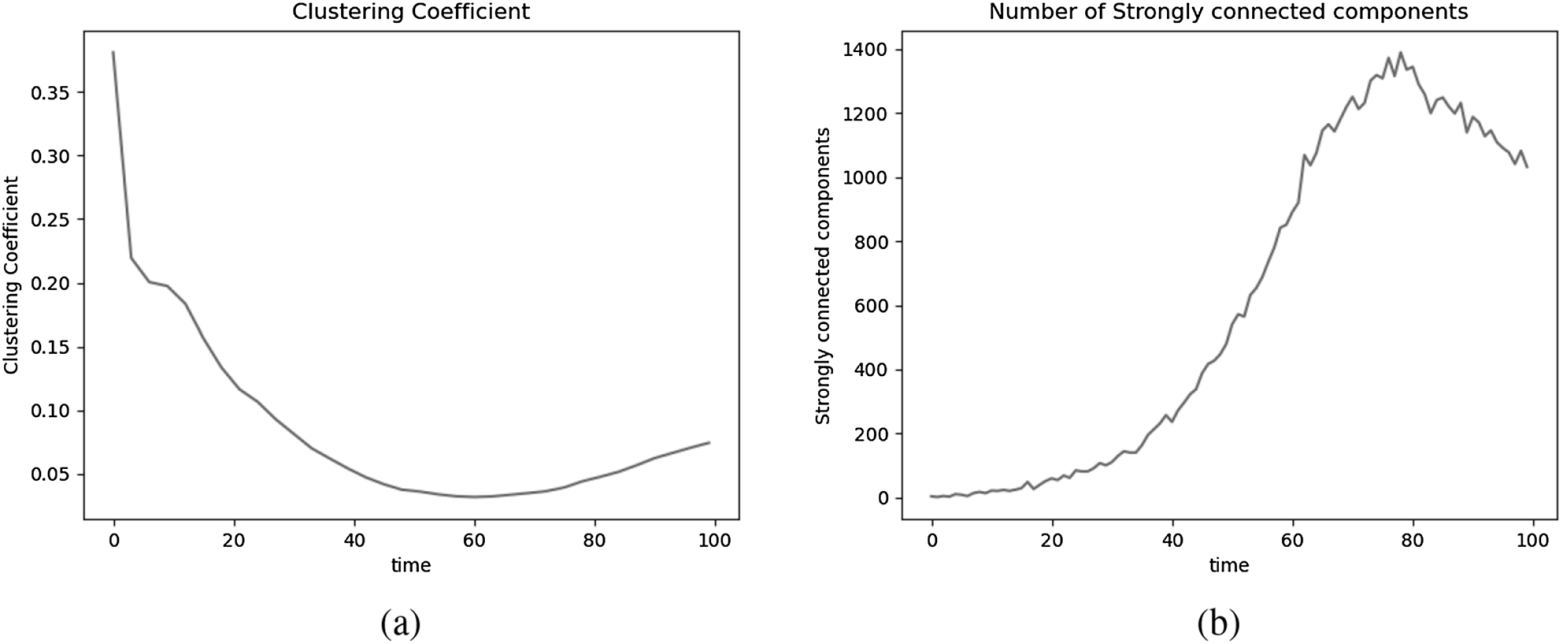
(**a**) clustering coefficient and (**b**) number of strongly connected components of directed networks generated using the selected model.

Using the same set of parameters as in the selected undirected model, we generated a series of networks that represents a directed online social network at different time-steps. Table 3 and Figs. 6 and 7 illustrate the results of executing the framework for directed networks. The properties of the network related to the number of accounts and the structure of the communities are similar to those of the undirected case, as the node and community updates are the same. The distribution of the number of followers and followings of the individuals is given in Fig. 6b, indicating that there are a few individuals with a large number of followers, as opposed to a large number of individuals with a relatively small number of followers, which is consistent with our hypothesis that there are a few famous individuals in the network. On the other hand, the number of followings ranges more narrowly and more closely resembles a normal distribution, which makes sense given that following an exceptionally large number of accounts is unusual.

**Table 3:** Properties of the generated networks of the selected directed model.

Parameter	Value
*N*	$5 \times 10^4$
$t^*$	100
$N_R^{t^*}$	18,836
Number of Edges	2,012,278
Average in/ out degree	106.8
Clustering coefficient	0.1

Our results indicate that the number of weakly connected components of the network always equals one, implying that the underlying undirected network is always connected. However, as illustrated in Fig. 7b, the network contains a large number of strongly connected components, making it impossible to compute the diameter of network accurately.

## Execution time

**Figure 8:**
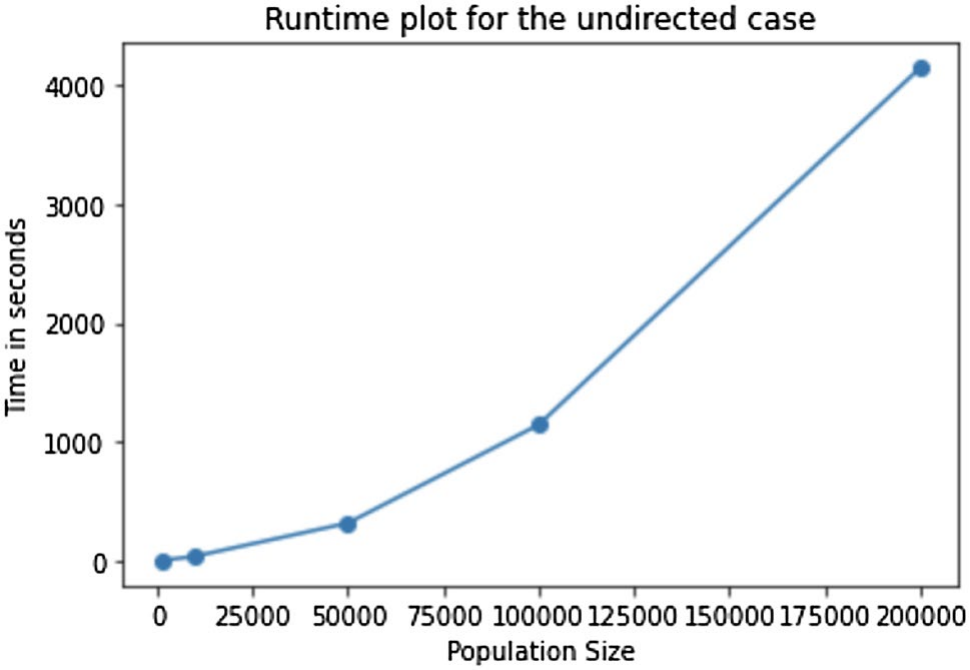
Comparison of the running times of the selected undirected model with different population sizes.

In this section, we discuss the run-time performance of the EpiCNet algorithm for undirected networks. The set of all tunable parameters other than the total population (*N*) are fixed as in Table 1. We varied *N* from $10^3$ to $2\times 10^5$ and measured the running time of the algorithm. The execution times for different population sizes are tabulated in Table 4 with the corresponding visual comparison in Fig. 8. We are able to fit a quadratic polynomial curve with $R^2 = 0.999$. All executions were done on a computer with 2.6 GHz 6-Core Intel Core i7 processor and 16 GB 2667 MHz DDR4 RAM.

**Table 4:** Running time of the selected undirected model for different population sizes.

Population size	Time (in seconds)
$10^3$	2.17
$10^4$	40.29
$5\times 10^4$	318.52
$10^5$	1144.38
$2 \times 10^5$	4143.31

## Conclusion

We identified the need to generate synthetic networks to parallel online social networks in terms of network properties. Accordingly, in this work, we developed an algorithm, EpiCNet, to generate a sequence of networks that evolve with time based on expected node behavior and community structure in social media platforms like Facebook. In a first, EpiCNet generates networks that evolve in a time-dependent manner by utilizing compartmental modeling from epidemiology and defining time-dependent variables that control the evolution of the networks. These properties of EpiCNet allows the generation of more realistic networks. The trends in standard network properties like clustering coefficient, network diameter, node degree, number and size of communities, and number of nodes in generated networks were validated to be consistent with the values established in various studies available in the literature.

Our simple and robust model evolves nodes, communities, and edges independent of any network properties. This evolution could be enriched to incorporate node attributes and domain specific node connection behaviors while creating edges. In addition, the running time of the model can be optimized to generate networks with a number of nodes at a scale parallel to that in real-world online social networks.

## Data Availability

Python implementation by the authors in addition to examples of the generated networks are available at https://github.com/pouyansh/EpiCNet.
